# Joint extension speed dictates bio-inspired morphing trajectories for optimal longitudinal flight dynamics

**DOI:** 10.1098/rsif.2023.0734

**Published:** 2024-04-24

**Authors:** C. Harvey

**Affiliations:** ^1^ Department of Mechanical and Aerospace Engineering, University of California, Davis, CA 95616, USA

**Keywords:** wing morphing, gliding flight, biomechanics, flight dynamics, flight control

## Abstract

Avian wing morphing allows dynamic, active control of complex flight manoeuvres. Previous linear time-invariant (LTI) models have quantified the effect of varying fixed wing configurations but the time-dependent effects of morphing between different configurations is not well understood. To fill this gap, I implemented a linear parameter-varying (LPV) model for morphing wing gull flight. This approach models the wing joint angles as scheduled parameters and accounts for nonlinear kinematic and gravitational effects while interpolating between LTI models at discrete trim points. With the resulting model, I investigated the longitudinal response associated with various joint extension trajectories. By optimizing the extension trajectory for four independent objectives (speed and pitch angle overshoot, speed rise time and pitch angle settling time), I found that the extension trajectory inherent to the gull wing does not guarantee an optimal response but may provide a sufficient response with a simpler mechanical implementation. Furthermore, the results indicated that gulls likely require extension speed feedback. This morphing LPV model provides insights into underlying control mechanisms, which may allow for avian-like flight in future highly manoeuvrable uncrewed aerial vehicles.

## 1. Introduction

It is challenging to define a comprehensive flight dynamics model that is valid and tractable across the complete flight envelope, even for traditional, fixed wing aircraft. This challenge is partly due to nonlinear aerodynamic, inertial and kinematic characteristics across standard flight conditions. For bird flight, this is further complicated by the geometrical and structural complexity of bird wings combined with wing morphing, a bird’s ability to dynamically change the shape of their wings in flight [1–3]. It is well established that birds adjust their wing configurations to adapt to different environmental conditions or to different flight modes [4–6], including pigeons gliding across various wind speeds [7] or falcons transitioning from level flight into a dive configuration (table 1) [8].

**Table 1.** Nomenclature.

***A***	state matrix
***B***	control matrix
***C***	output matrix
***D***	coupling matrix
***F***	force vector
*I_yy_*	moment of inertia about the *y*-axis
*M*	absolute aerodynamic moment about the *y*-axis
*Q*	pitch rate
*U*	velocity in the *x*-axis
***U***	control vector
*W*	velocity in the *z*-axis
*X*	absolute aerodynamic force in the *x*-axis
***X***	state vector
***Y***	output vector
*Z*	absolute aerodynamic force in the *z*-axis
*c_max_*	maximum root chord
*f*	frequency of the extension and fold cycle
*g*	gravitational acceleration
*k*	iteration step
*k_aero_*	reduced frequency
*m*	bird mass
*q*	perturbation in pitch rate
*u*	perturbation in *x*-axis velocity
*w*	perturbation in *z*-axis velocity
*x*	body *x*-axis, positive pointing out of the beak of the bird
*y*	body *y*-axis, positive pointing out of the right wing of the bird
*z*	body *z*-axis, positive pointing downwards from the bird
*t*	time
δ_e_	elbow angle
δ_w_	wrist angle
Θ	pitch angle
***ρ***	scheduled parameter vector

Birds morph their wings actively by manipulating their shoulder, elbow, wrist or digit joint angles [9,10]. The kinematics of this shape change is complex and three-dimensional, and is often approximated as a six-bar linkage [11]. Because of this coupled linkage system, bird wings exhibit an underactuated response when force is applied solely to the wrist joint with a fixed shoulder joint [12]. This single input causes the extension of both the elbow and wrist angles and is referred to within this work as the *linkage extension trajectory*. This underactuated system is expected to yield a simpler and, possibly, more energy-efficient way to extend the wing. In a previous study, Baliga *et al*. determined the linkage trajectory for over 60 bird species, including the gull species used in this work, and found that flight behaviour was a key explanatory variable for trajectory differences between species [12].

However, avian flight dynamics are rarely investigated, often because of the many degrees of freedom of the biological system as well as the associated nonlinearities. Without this information, it is difficult to identify which unique attributes of bird flight best facilitate the development of a new generation of more manoeuvrable bio-inspired uncrewed aerial vehicles (UAVs). To fill this gap, engineering tools can be employed to model complex animal flight behaviours.

Recent studies on morphing aircraft, including UAVs, have implemented linear parameter-varying (LPV) methods to model the dynamics associated with morphing between different wing configurations [13–18]. The LPV methodology provides a first-order prediction of the dynamic response of a morphing wing flyer by effectively interpolating between linear time-invariant (LTI) models defined at known trim states across the region of interest. A trim state is an equilibrium state where the flyer produces positive lift to provide weight support. LPV models traditionally use look-up tables to bridge discrete trim states while accounting for nonlinear kinematic and gravitational characteristics [13,14]. Note that it is currently not known when, or if, birds fly in a trim state as much of their flight manoeuvres are transient behaviours that may not require the existence of an equilibrium condition. This current work provides initial insight into the flight dynamics associated with morphing in a trimmed flight condition, which is most likely to be relevant for long-range gliding behaviours.

A bird’s complete range of motion is known to provide the capacity to shift between longitudinal static stability states [19], although it is not yet known if or when birds actively adjust between these states. Manipulating the elbow and wrist joints also allows gulls to substantially adjust their dynamic stability characteristics [20]. Previous work derived the rigid body longitudinal dynamics at known trim states across a regularly spaced grid of elbow, wrist and shoulder angles by calculating the stability derivatives for each wing configuration [20]. By assuming symmetric flight conditions and that birds experience only small disturbances from the trim states, LTI models were obtained for each combination of wing joint angles. These LTI models are useful for discussing the dynamics of the gull in each discrete fixed wing configuration, however, they are unable to account for the transition between different wing joint angles.

Here, I build on these advances to implement an LPV model that quantifies the flight dynamics of a gliding hybrid glaucous-winged and western gull (*Larus glaucescens* × *occidentalis*), also known as an Olympic gull, during wing morphing. This work focuses on the longitudinal response of the bird due to morphing between two distinct longitudinally stable wing configurations following scheduled elbow and wrist extension trajectories.

## 2. Methodology

### 2.1. Implementing the linear parameter-varying model

**Figure 1.**
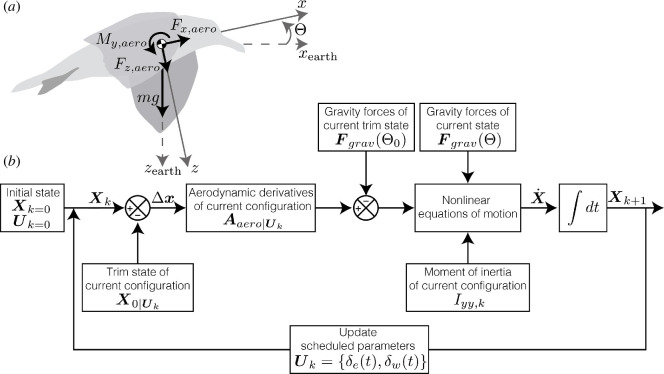
Simulation model of a gliding gull during scheduled wing morphing. (*a*) Free body diagram of the gull model. (*b*) Block diagram implementation of the linear parameter-varying model for a morphing wing gull. All symbols are defined within the below nomenclature and text.

This work uses a gull model with physical parameters based on biological measurements from gull cadavers [20] to establish a dynamic simulation that incorporates aerodynamic and inertial forces (figure 1*a*). The elbow and wrist were selected to be scheduled morphing parameters and the shoulder angle remained fixed with a 5° forward sweep and 20° dihedral angle. This fixed position was selected by identifying the shoulder position with the highest number of trim states for the given extension trajectory (see §3.2. for details). This fixed shoulder angle assumption simplifies the model implementation, although future work could incorporate a scheduled shoulder angle as well. The modelled bird had a mass of 1.015 kg and the moment of inertia about the pitch axis ($I_{yy}$) varies from 0.0047 to 0.0086 kg·m^2^ due to the range of motion of the elbow and wrist angle at the fixed shoulder angle (refer to table 1 for nomenclature). The inertial characteristics were calculated using *AvInertia,* a computational method based on a classical mechanics approach to approximate the centre of gravity and moment of inertia of a bird in a given wing configuration [19]. The maximum wing root chord (0.286 m) and the maximum wing and body area (0.268 m^2^) were used as the reference length and area, respectively, and were held constant throughout morphing. A furled (folded) tail at 0° incidence was included in the model based on cadaver tail measurements and a NACA 0006 airfoil [20].

The stitched model architecture developed by Tischler & Tobias motivated the implementation of the current LPV model [14]. The longitudinal mode was decoupled from the lateral modes, assuming small manoeuvres and symmetric flight conditions. Note that Tischler & Tobias’ approach is based on a quasi-linear parameter-varying model (qLPV) that is stitched between known states of the system, such as the speed [14]. However, as the current work’s focus is to analyse the gull flight dynamics due to wing morphing, the system states were not included as stitched parameters in this analysis.

A general LPV model can be written as a linear state-space model that assumes the following form:


(2.1) $$\left[\begin{array}{c} \dot{X}\\ Y \end{array}\right]=\left[\begin{array}{cc} A(\rho (t)) & B(\rho (t))\\ C(\rho (t)) & D(\rho (t)) \end{array}\right]\left[\begin{array}{c} X\\ U \end{array}\right].$$

Where ***A***, ***B***, ***C***, ***D*** represent the state-space matrices, ***X*** is the state vector, ***U*** is the control vector and ***Y*** is the output vector. Note that vectors and matrices are represented by boldface symbols and scalars are represented by non-boldface symbols. The state-space matrices are defined as a function of ρ(t), the time-dependent scheduled parameter vector, which includes the elbow ($\delta_{e}$) and wrist ($\delta_{w}$) angle for the current time step, k. As this study schedules both the elbow and wrist angle at each time step, the parameter vector equals the control vector, $U_{k}$***,*** at each time step as:


(2.2) $$\rho (t)=\left[\begin{array}{c} \delta_{e}(t)\\ \delta_{w}(t) \end{array}\right]=U_{k}.$$

The time-dependent functions of the elbow and the wrist angle are discussed in further detail in §3.2.

The architecture visualized in figure 1*b* was implemented in a custom Python code. First, the trim state vector ($X_{0}$) was determined for the current wing configuration ($U_{k}$) as:


(2.3) $$X_{0|U_{k}}={\left[\begin{array}{c} U_{0}\\ W_{0}\\ Q_{0}\\ \Theta_{0} \end{array}\right]}_{|U_{k}}.$$

Trim state variables are represented by a 0 subscript. The trim state vector ($X_{0}$) in a longitudinal analysis includes the trim speed in the body *x*-axis ($U_{0}$), the trim speed in the body *z*-axis ($W_{0}$), the trim pitch rate ($Q_{0}$) and the trim pitch angle ($\Theta_{0}$). This information was accessible from previous work that had calculated the trimmed states of a gull on a regularly spaced grid of varied elbow and wrist angles at a few fixed shoulder angles using the aerodynamic coefficients for lift, drag, and pitching moment and the inertial characteristics associated with each configuration [20]. The aerodynamic coefficients were estimated using a numerical implementation of a general lifting-line method (MachUpX). This low-fidelity model was experimentally validated for nine different gull wing configurations at low angles of attack [21–23].

Next, to interpolate between the known trim states for both the elbow and wrist angle, linear models were fit to the estimated aerodynamic coefficients based on previously established polynomial relationships between the elbow and wrist angle [20]. These linear aerodynamic predictions replaced the look-up tables that are traditionally used in LPV methods. Next, the identified trim state vector was subtracted from the current state ($X_{k}$) to define the perturbation state vector (∆x). For this implementation, it is not necessary to calculate the perturbation control vector since the entire control vector was scheduled (equation (2.2)), and thus the effects of the joint angles are contained implicitly in the changes of the stability derivatives between states as proven analytically by previous studies [14,16].

Next, the aerodynamic stability derivatives associated with the current wing configuration were extracted to form the aerodynamic-specific, state-space matrix ($A_{aero|U_{k}}$) as:


(2.4) $$A_{aero|U_{k}}={\left[\begin{array}{ccc} X_{u} & X_{w} & X_{q}\\ Z_{u} & Z_{w} & Z_{q}\\ M_{u} & M_{w} & M_{q} \end{array}\right]}_{|U_{k}}.$$

Each component of this matrix is a derivative of the aerodynamic forces (*X, Z*) and moments (*M*) with respect to speed (*u, w*) and pitch rate (*q*) perturbations. These perturbation stability derivatives are defined within the body axes rather than the traditional stability axes as this simplifies the inclusion of nonlinear gravitational effects [14]. This matrix is multiplied by the perturbation state vector (∆x) to obtain the perturbation forces and moments ($∆F_{x,aero},∆F_{z,aero},∆M_{y,aero}$). This step is necessary as the current architecture calculates the total forces rather than non-dimensional coefficients [14].

Note that the mass (*m*) and moment of inertia (*I_yy_*) have not entered the equations at this point. To incorporate the inertial effects, the nonlinear gravitational forces calculated at the trim state are subtracted and those calculated at the current state are added to the equation. This approach enforces that, when the current state is a trim state for the current wing configuration, these two contributions will offset each other. Finally, the nonlinear equations of motion are formulated for the current state allowing for nonlinear kinematics as follows:


(2.5) $$\left[\begin{array}{c} \dot{U}\\ \dot{W}\\ \dot{Q}\\ \dot{\Theta } \end{array}\right]=\left[\begin{array}{c} -QW+(\Delta F_{x,aero}+mg\sin\Theta_{0}-mg\sin\Theta )/m\\ QU+(\Delta F_{x,aero}-mg\cos\Theta_{0}+mg\cos\Theta )/m\\ \Delta M_{y,aero}/I_{yy}\\ Q \end{array}\right].$$

Using the Python function *odeint,* these equations were integrated in time for one-time step (*dt* = 0.005 s) to extract the new state values ($X_{k+1}$) that were used for the next iteration. At this stage, the code updates the elbow and wrist angle to the next scheduled state and calculates the new centre of gravity in this configuration.

This methodology accounts for a variable centre of gravity and moment of inertia but assumes that the bird’s mass remains constant. Because the LTI models that informed the aerodynamic state matrix were each defined with the aerodynamic moment taken about the instantaneous centre of gravity, the effect of the variable centre of gravity on the aerodynamic moment is implicitly accounted for within each time step. Note that this method does not include dynamics associated with the acceleration of the centre of gravity between morphed configurations. This is expected to be negligible due to the slow extension speeds tested as well as because of the small total centre of gravity motion. Across all trim states, the largest shift in the centre of gravity is 2.7 cm along the body *x*-axis and 0.9 cm along the body *z*-axis, where the gull torso length is approximately 45 cm. To account for variation in the moment of inertia between configurations, each time step’s nonlinear equations of motion were solved using the moment of inertia of the current wing configuration following the procedure detailed by Tischler & Tobias [14].

### 2.2. Scheduling the joint extension trajectories

**Figure 2.**
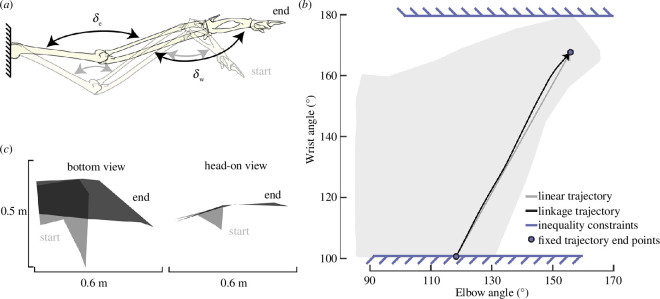
The linkage extension trajectory arises due to coupling between the elbow and wrist angles when force is applied to the wrist joint. The (*a*) joint angles and (*c*) wing planforms shown approximately represent the start and end wing configurations. Adapted from [21]. (*b*) The scheduled linkage extension trajectory (black line) and linear trajectory (grey line) are overlaid on the range of motion of the gull. The optimization routine held the end points fixed and imposed inequality constraints visualized by the blue lines.

With the LPV model architecture, it is necessary to schedule the elbow and wrist joint angles as a function of time. The full two-dimensional parameter space defined by a gull’s elbow and wrist angle can be considered for scheduling these joints (figure 2*c*) [21]. To narrow the focus of this study, the linkage extension trajectory, that is, the extension of the bird wing skeleton linkage due to a force applied on the wrist, was investigated (black line in figure 2*c*). The starting configuration has an elbow angle of 120.7° and a wrist angle of 105.3° and the ending configuration has an elbow angle of 155.8° and a wrist angle of 167.7° (figure 2*a, b*). These angles are within ranges known to be used by gliding gulls in flight [6]. At a fixed shoulder angle of 0° sweep and 0° dihedral angle, this trajectory produces variable and high magnitude changes in the lift and pitching moment. For example, near the beginning of the trajectory (lower wrist angles), the coefficient of lift increases with the extension, whereas near the end of the trajectory (higher wrist angles), the coefficient of lift reduces with extension [21].

Extension between the start and end configurations shifts the bird from a trimmed high-speed dive at 33 m s^−1^ into a slower (17 m s^−1^) trimmed glide with a pitch angle of 29.2°. These trimmed states are computed for the selected start and end elbow and wrist configurations rather than corresponding to conditions known to be used by gulls. The low speeds are within ranges known to be used by gulls in a glide [24], but there is little information on the speed of a gull in a diving condition. Both configurations are stable, allowing this approach to solely consider the open-loop dynamics of the bird.

**Figure 3.**
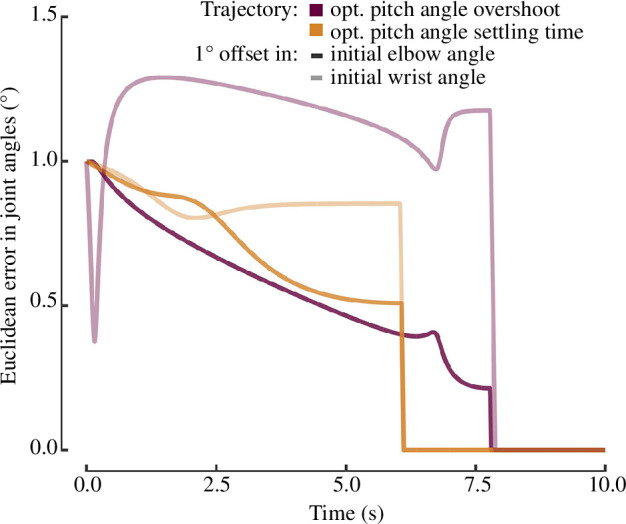
Sensitivity of the optimized trajectories to the initial joint configuration.

The dynamic response associated with the linkage trajectory was first compared to that of a linear path between the same start and end points (grey line in figure 3). The linear trajectory is the most direct path between these two configurations and has only minor deviations from the linkage extension trajectory, where the largest difference in the wrist angle for a given elbow angle is 3.7°. To create this exact linear extension path in a gull wing, independent control of both the elbow and wrist joint may be required. However, this difference is within a range that may be explained by experimental error in the initial linkage analysis [6]. Therefore, these two trajectories are considered to determine the sensitivity of the dynamics model to slight trajectory variations.

The extension along these two trajectories was compared at three different extension rates: 4, 8 and 12° s^−1^. These rates simulate a slow morphing response in a gliding configuration, unlike the faster morphology changes that would occur in flapping flight, where gulls have a wing flapping frequency in the range of 3–4 Hz [25]. Future work is required to identify if these morphing extension speeds are biologically relevant within a gliding configuration. These slow extension speeds were selected to minimize the role of unsteady aerodynamic effects, which were not accounted for within this work. The degrees used to define the extension speed were calculated based on the Euclidean distance between the elbow and wrist angles along each trajectory as:


$$extension\;distance=\;\sqrt{\delta_{e}^{2}+\delta_{w}^{2}}.$$

To validate the quasi-steady assumption, a reduced frequency, $k_{aero}$ , can be computed based on the time to extend and fold (i.e. one cycle) as:


$$k_{aero}=\;\frac{fc_{max}}{2U_{min}}.$$

Where f is the frequency of the complete cycle, $c_{max}$ is the maximum chord at the root and $U_{min}$ is the minimum gliding speed across the extension (17 m s^−1^). These parameters were selected to give insight into the highest possible reduced frequency across the morphed states. This reduced frequency was below 0.00075 for all extension speeds, which confirms the appropriateness of the quasi-steady assumption.

Next, an optimization routine was implemented to determine extension trajectories that would optimize the dynamic response characteristics between the start and end configurations. Within this framework, four key objective functions were separately minimized: speed overshoot, pitch angle overshoot, speed rise time and pitch angle settling time. In this work, the parameters are defined as follows: overshoot is the relative difference between the absolute maximum time series value and the final settled value, settling time is the time taken until the relative difference between an oscillation peak and the final settled value is only 2% of the final settled value, and rise time is the time taken until the relative difference is under 2% of the final settled value for the first time. These objectives were chosen to represent desirable flight responses. Each objective function was minimized for the three extension rates independently, resulting in 12 possible optimal trajectories.

The optimization routine used the SciPy sequential least squares programming (SLSQP) algorithm [26]. The optimized variables were two of the four coefficients (c_2_, c_3_) of a third-order polynomial:


$$\delta_{w}=c_{0}+c_{1}\delta_{e}+c_{2}\delta_{e}^{2}+c_{3}\delta_{e}^{3}.$$

The other two coefficients were calculated within the routine directly to enforce that the end and start point remained fixed (circles, figure 2*c*). Inequality constraints were implemented to ensure that the maximum wrist angle was never greater than the maximum biologically possible wrist angle (~179°) and so that the minimum wrist angle was never less than 105° to stay within the same range used by the previous aerodynamic studies (blue lines, figure 2*c*). The inequality constraints were implemented by selecting 20 points along the trajectory and imposing the minimum or maximum constraint depending on their proximity to the start or end, respectively. Points were clustered around the start and the end of the trajectory. Note that the use of a polynomial function with a dependency on elbow angle enforces a monotonic increase of the elbow angle. In addition, broad bounds were imposed on the optimized variables (−1000 $<c_{2}<$1000 and −50 000$<c_{3}<$ 50 000) to reduce the chance that the optimizer took steps into regions that violated the dynamics model. For cases where the optimizer took steps that violated the dynamics model, a sum of all wrist angles was returned. For each case, a linear trajectory ($c_{2}=0$, $c_{3}=0$) was used as the initial guess. The SLSQP default convergence criteria were used, and all solutions converged. All final polynomial coefficients are reported in table 2.

**Table 2.** Polynomial coefficients for each extension trajectory.

**e**xtension speed (°/s)	**o**bjective function	$c_{0}$	$c_{1}$	$c_{2}$	$c_{3}$
-	linear	−109.2	1.78	0	0
-	linkage	1211.5	−27.97	0.222	−0.0005
4	opt. pitch angle overshoot	5436.5	−116.03	0.828	−0.0019
4	opt. speed overshoot	5441.9	−116.15	0.829	−0.0019
4	opt. pitch angle settling time	−5175.6	117.14	−0.870	0.0022
4	opt. speed rise time	−6715.7	152.94	−1.146	0.0029
8	opt. pitch angle overshoot	12609.3	−280.20	2.073	−0.0050
8	opt. speed overshoot	12542.1	−278.66	2.061	-0.0050
8	opt. pitch angle settling time	−5734.0	129.65	−0.963	0.0024
8	opt. speed rise time	−6522.9	148.12	−1.106	0.0028
12	opt. pitch angle overshoot	15763.4	−352.47	2.621	−0.0064
12	opt. speed overshoot	15767.1	−352.56	2.622	-0.0064
12	opt. pitch angle settling time	−6881.5	154.54	−1.142	0.0028
12	opt. speed rise time	−7390.4	166.58	−1.236	0.0031

The sensitivity and convergence of the optimization routine were investigated. First, the effect of the size of the time step (*dt*) used in the dynamic solution was explored by determining the optimal trajectory for a time step of 0.001 s, 0.0025 s and 0.005 for the test case of minimizing the pitch angle overshoot at an extension of 12° s^−1^. There were no identifiable differences in the optimal trajectories for each case, therefore the largest time step (0.005 s) was selected to reduce the computational time. Next, the sensitivity of the optimal trajectory to the starting position was evaluated. The elbow and wrist angle were perturbed by 1° for two objective functions and minimal changes in the optimal trajectories were obtained (figure 3). For all cases, the Euclidean error of the wrist and elbow angles never exceeded 1.5°. Finally, all optimization cases terminated successfully with 74 iterations or less.

## 3. Results and discussion

### 3.1. Case study 1: biologically relevant linkage trajectory versus linear trajectory

**Figure 4.**
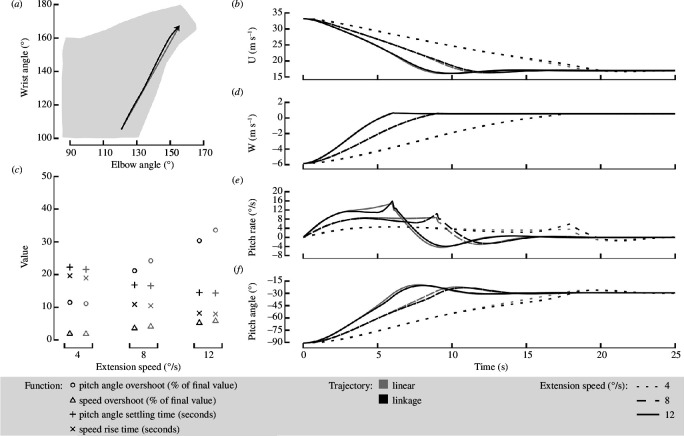
There was minimal impact on the dynamic response of a gliding gull due to wing extension along the linkage (black) or linear (grey) trajectory. (*a*) The two extension trajectories plotted over the gull’s complete range of motion. (*b*,*d*–*f*) The time response of each component of the state vector for an extension of 4° s^−1^ (dotted line), 8° s^−1^ (dashed line), 12° s^−1^ (solid line). (*c*) Four key dynamic parameters were investigated: the speed and pitch angle overshoot shown as a percentage of the final settled value as well as the speed rise time and pitch angle settling time shown in seconds. Extension was considered at three different speeds.

First, I investigated the differences between the linkage trajectory (black line) and the linear trajectory (grey line) to examine the potential utility of a biologically relevant extension and the effect of slight variations on the dynamic response. As expected, due to the similarity between the trajectories, I found only minor variations in the dynamic responses at all three investigated speeds (figure 4). Of note, the linear trajectory had a steadier increase in the pitch rate at higher extension speeds resulting in a softer peak (solid grey line in figure 4*e*), although the following oscillatory response is nearly indistinguishable from the linkage trajectory. To quantify the variation between these trajectories, I calculated the pitch angle overshoot, speed overshoot, pitch angle settling time and the speed rise time (figure 4*b*). The largest percentage difference in any metric was the 13% increase in the overshoot of the pitch angle and speed for the linear trajectory (grey circles and triangles, figure 4*b*).

These results indicate that the resulting LPV model is not highly sensitive to the exact joint angle inputs. Lower sensitivity to small variations in the controls (i.e. the joint angles) will allow for some experimental uncertainty and manufacturing variability associated with attaining an exact targeted joint angle. This outcome provides confidence that this model could effectively be used to develop a controller and simplify its implementation on an avian-inspired morphing UAV.

For both trajectories, the extension speed had a notable effect on the dynamic response, even though unsteady aerodynamic effects are not considered in this work (figure 4*b*). As expected, increasing the extension speed increased overshoot, and reduced the rise time and settling time for both trajectories. However, I also found that for the two faster extension speeds (8 and 12° s^−1^), the linear trajectory had a larger overshoot in speed and pitch angle than the linkage trajectory, whereas at 4° s^−1^ this trend reversed (figure 4*b*).

### 3.2. Case study 2: trajectories to optimize the longitudinal dynamic response

Next, I investigated if different extension trajectories could optimize the dynamic response between the start and end wing configurations. Given that the longitudinal dynamic response is characterized by multiple parameters, I specifically considered four objective functions that were expected to be important for effective flight control of a UAV: minimal speed overshoot, pitch angle overshoot, speed rise time and pitch angle settling time.

**Figure 5.**
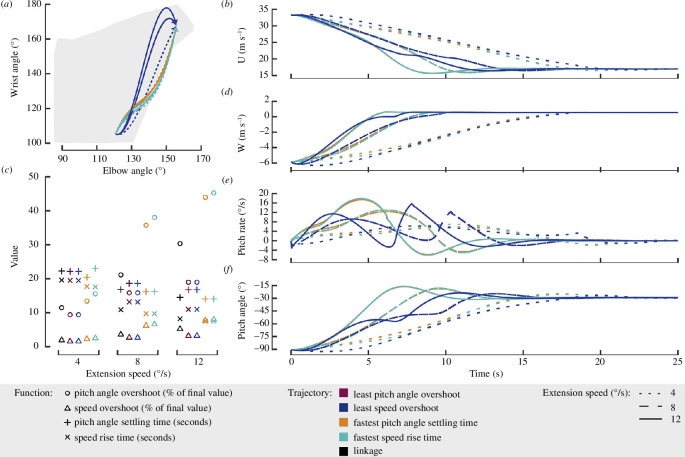
The optimal dynamic response of a gliding gull extending its wing depends on the objective function and extension speed. (*a*) The optimal extension trajectories for each objective function at each speed (12 in total) are plotted over the gull’s complete range of motion. (*b,d–f*) The time response of each component of the optimal trajectories state vector for an extension of 4° s^−1^ (dotted line), 8° s^−1^ (dashed line), 12° s^−1^ (solid line). (*c*) The four key dynamic parameters were investigated: the speed and pitch angle overshoot shown as a percentage of the final value as well as the speed rise time and pitch angle settling time shown in seconds. Extension was considered at three different speeds. These parameters are compared to the linkage results from figure 4*b* (black symbols).

The trajectories that minimized the speed overshoot (dark blue line, figure 5) and the pitch overshoot (maroon line, figure 5) had a strong dependence on the extension speed. The optimal trajectory for minimizing the overshoot in both speed and pitch angle resulted in nearly the same extension trajectory, as the maroon line lies underneath the dark blue line (figure 5*a*). At the lowest speed (dotted lines), the optimal trajectory resembles the linkage trajectory. However, as extension speeds increase, the optimal wrist angle overshoots the final configuration allowing a smoother transition in the speed and the pitch angle. These trajectories cause a ‘step-like’ response in all four degrees of freedom that becomes more pronounced at higher extension speeds (figure 5*c–f*). Together, these results again suggest that the extension speed plays an important role in the control gained through wing joint extension.

The trajectories that minimized the speed rise time (teal line, figure 5) and the pitch angle settling time (orange line, figure 5) were notably similar and there was minimal effect of extension speed on the optimal extension trajectory (figure 5*a*). Unlike the overshoot optimal trajectories, these extension trajectories have a smoother pitch rate peak (figure 5*e*). As a result, a higher pitch rate was developed for a longer period of time within each extension, likely playing a key role in minimizing the rise and settling time. In both cases, the trajectories slightly departed from the biologically relevant range (grey shading figure 5*a*) due to a quick increase in wrist angle followed by an increase in the elbow angle near the beginning of the trajectories. Again, note that there is experimental error associated with estimating the bird wing joint angles on the order of a few degrees [6]. Therefore, it is possible that a live gliding gull could obtain trajectories that only slightly diverge from the shaded region.

Next, I compared these optimal trajectories to the biologically relevant linkage trajectory (figure 5*b*). At low speeds, there was a minimal improvement when comparing the optimal trajectory for each objective functions to the linkage trajectory. However, as the speeds increase, it becomes more favourable to switch to an optimized trajectory, especially to reduce the pitch angle overshoot noted by larger gaps between the relevant objective functions (blue and maroon open circles) and the linkage trajectory (black open circles). Across all conditions, the dynamic response of the linkage system had only slight differences in all of the four key objective functions compared to any of the optimal solutions. Therefore, it is possible that the linkage trajectory may allow multiple flight objectives to be balanced. Exploring this hypothesis with a multi-objective optimization analysis will be an important next step for evaluating the value added by using the linkage extension trajectory.

In all, each trajectory provides a different method to achieve the same end condition, although the time-dependent response of the bird relies on the trajectory that is followed. For applications requiring a quick reduction in speed, the trajectory that has the lowest rise time would be preferable (figure 5*b*, teal symbols). However, to minimize energy usage or simplify actuation design, it may be best to use a linkage extension trajectory, since there is a single point of actuation, and it suffers only a 9–12% slower rise time compared to the optimal trajectory depending on extension speed (figure 5*b*, black symbols). These differing optimal trajectories suggest that the flight control algorithm used by birds must be informed by the relationship between the joint extension speed and the resulting flight dynamics. It is likely that the extension-speed-dependent controller would also need to account for dependencies associated with unsteady aerodynamic effects.

In this work, I aimed to establish an initial approach to investigate the dynamic response associated with wing morphing in bird flight and allow for further design and biological complexity to be built upon these foundations. Many simplifications and assumptions were necessary to develop these equations. The effects of wing flexibility are neglected as previous work found minimal effect of avian-inspired flexibility on the aerodynamic forces of an airfoil within Reynolds numbers that are relevant to birds [27]. How feather flexibility affects avian flight controls should be investigated in future work. Aerodynamic characteristics were estimated using quasi-steady assumptions and neglected lateral contributions because this work focuses on morphing within longitudinal gliding flight. The quasi-steady assumption was verified by characterizing the reduced frequency associated with the tested extension speeds. However, to analyse any sudden, fast manoeuvres performed by birds, a coupled lateral–longitudinal model will be required. Note that it is possible that the extension speed may affect the wing’s morphology due to differences in the soft tissue properties and elastic energy storage. The differences between cadaveric measurements and live birds will be important to decipher.

Although the shift in the centre of gravity and moment of inertia is included by calculating the aerodynamic moment at each discrete trim state, this work neglects additional terms associated with a moving centre of gravity due to the slow morphing and small absolute shifts [28]. Future work is required to test this assumption for high-speed bird flight manoeuvres. In addition, constraints were not imposed to keep the optimal path within the biologically relevant range. This work could be reframed to remove the cubic trajectory assumption and instead constrain each trajectory point to remain within the grey-shaded area shown in figure 2*c*.

Finally, the existence of a trim state is fundamental to this stability analysis, however, there is little current evidence of birds flying in a trimmed condition. Many bird manoeuvres are likely transient states and thus, in future work, it will be crucial to explore these mathematical stability results in contrast with live recordings of birds to ensure that the proper flight dynamics are being captured. This will also allow the computed optimal trajectories to be directly compared to the extension trajectories that birds use to morph their wings in flight.

## 4. Conclusions

This study investigated the longitudinal flight dynamics associated with extending the gull wing between two wing configurations. First, the biologically relevant linkage trajectory was found to provide a similar dynamic response to a linear extension trajectory, which highlights the dynamic response is not highly sensitive to the input joint angles. This is promising for the integration of such a mechanism on a future UAV. Distinct optimal trajectories were found that could be separated in two similar cases: (1) minimizing the speed and pitch angle overshoot and (2) minimizing the speed rise time and the pitch angle settling time. The trajectories that minimized overshoot in speed and pitch angle varied substantially as the joint extension speed was varied. This suggests that to control their flight when considering overshoot characteristics, birds would need to process information relating to their joint extension speed for effective closed-loop flight control. Although not optimal, the dynamic response of the linkage system had only minor differences in the four key objective functions compared to the optimized results. This suggests that investigating a multi-objective optimization in the future could be insightful for this biological system.

In all, this work adapts a flight dynamics approach used to model aircraft flight dynamics across discrete states to investigate the flight dynamics of a gliding bird during wing morphing. Birds provide regular evidence that morphing wings play a substantial role in highly manoeuvrable and adaptable flight. The present work takes a necessary step towards understanding avian flight control by providing an initial quantification of the longitudinal flight dynamics associated with dynamic joint extension for gliding birds. The results highlight the importance of understanding the control inputs and feedback signals required for birds to actively control their complex flight manoeuvres to advance true avian-like manoeuvrable flight in the next generation of UAVs.

## Data Availability

All data and codes reported in this article have been deposited in the Figshare public repository [30].
